# Management of neurological complications of infective endocarditis in ICU patients

**DOI:** 10.1186/2110-5820-1-10

**Published:** 2011-04-20

**Authors:** Romain Sonneville, Bruno Mourvillier, Lila Bouadma, Michel Wolff

**Affiliations:** 1 Service de Réanimation Médicale et des Maladies Infectieuses, EA 3964, Université Paris 7-Denis Diderot, Hôpital Bichat-Claude Bernard, 46, rue Henri-Huchard, 75877 Paris Cedex 18, EA 3964, University Paris 7, France

## Abstract

Patients with infective endocarditis (IE) are generally referred to the intensive care unit (ICU) for one or more organ dysfunctions caused by complications of IE. Neurologic events are frequent causes of ICU admission in patients with IE. They can arise through various mechanisms consisting of stroke or transient ischemic attack, cerebral hemorrhage, mycotic aneurysm, meningitis, cerebral abscess, or encephalopathy. Most complications occur early during the course of IE and are a hallmark of left-sided abnormalities of native or prosthetic valves. Occlusion of cerebral arteries, with stroke or transient ischemic attack, accounts for 40% to 50% of the central nervous system complications of IE. CT scan is the most easily feasible neuroimaging in critically unstable patients. However, magnetic resonance imaging is more sensitive and when performed should follow a standardized protocol. In patients with ischemic stroke who are already receiving oral anticoagulant therapy, this treatment should be replaced by unfractionated heparin for at least 2 weeks with a close monitoring of coagulation tests. Mounting evidence shows that, for both complicated left-sided native valve endocarditis and *Staphylococcus aureus *prosthetic valve endocarditis, valve replacement combined with medical therapy is associated with a better outcome than medical treatment alone. In a recent series, approximately 50% of patients underwent valve replacement during the acute phase of IE before completion of antibiotic treatment. After a neurological event, most patients have at least one indication for cardiac surgery. Recent data from literature suggest that after a stroke, surgery indicated for heart failure, uncontrolled infection, abscess, or persisting high emboli risk should not be delayed, provided that the patient is not comatose or has no severe deficit. Neurologic complications of IE contribute to a severe prognosis in ICU patients. However, patients with only silent or transient stroke had a better prognosis than patients with symptomatic events. In addition, more than neurologic event *per se*, a better predictor of mortality is neurologic dysfunction, which is associated with location and extension of brain damage. Patients with severe neurological impairment and those with brain hemorrhage have the worse outcome.

## Introduction

The demographic characteristics of patients who develop infectious endocarditis (IE) have changed during the past few decades. Today, patients tend to be older, their underlying diseases have changed, *Staphylococcus aureus *has emerged as a predominant causative organism, and there is an increasing incidence of health care-associated infections. The exact proportion of patients with IE requiring admission to the ICU (except those admitted after cardiac surgery) is unknown. IE is associated with a myriad of complications, both cardiac and extracardiac, which may require ICU admission. Local progression of the infection causes destruction of valve cusps or leaflets and chordae and may extend to peri- and paravalvular structures. Hemodynamic deterioration leads to secondary organ failure. Finally, embolization of infected tissues may damage vital organs and cause peripheral abscesses. Intensivists often are confronted with complex treatment decisions regarding management of these complications. Neurologic complications are a frequent cause of ICU admission in patients with IE and are generally accepted as major determinants of poor prognosis with increased morbidity and mortality. The goal of the present review was to summarize current data on the incidence, mechanisms, clinical patterns, and consequences on outcome of critically ill patients with neurologic complications of IE.

## Incidence

The incidence of neurologic events during the course of IE varies greatly among series, tending to be higher in those gathered from referral centers. Because they contribute to death in IE, ancient studies based on autopsies and performed during the 1960s revealed brain lesions in up to 90% of patients [1]. In most series, central nervous system (CNS) involvement during the course of IE occurs in 20% to 40% of cases. Among 1,329 episodes of IE from seven series described between 1985 and 1993, 437 (33%) were accompanied by CNS manifestations [2]. In a Finnish teaching hospital, 55 of 218 IE (25%) were associated with neurologic complications [3]. However, in series published after 2000, the incidence of neurologic complications is lower: in France, strokes occurred in 17% of 264 IE cases caused by staphylococci or streptococci [4]; in the United States, among 513 episodes of complicated, left-sided native valve IE, focal neurologic signs or altered mental status were observed in 18% and 16% of cases, respectively [5]. Experience from the large, contemporary, International Collaboration on Endocarditis-Prospective Cohort Study (ICE-PCS) involving 2,781 patients from 58 hospitals in 25 countries reported a similar (17%) incidence of strokes [6]. Because neurologic events are a frequent cause of admission to the ICU of patients with IE, the percentage of this complication is higher in critically ill patients. Among 228 episodes of IE in 2 ICUs, neurological events were the most frequent complications, occurring in 37% of the patients [7]. A recent multicenter study showed a 55% incidence of neurologic events (mostly symptomatic) among 198 critically ill patients with left-sided endocarditis (Table 1). However, the true incidence of neurologic complications is difficult to assess because few studies used systematic neuroimaging. Those in which CT scan was performed for all patients [8,9] have shown that the CNS is more frequently involved in patients with IE than neurologic symptoms would suggest. Moreover, when cerebral magnetic resonance imaging (MRI) is systematically performed, cerebral lesions are found in at least 80% of the patients, most having no neurologic symptoms [10-12]. Therefore, the overall incidence of brain complications appears to be much higher than that detected by previous clinical studies.

**Table 1:** Neurologic complications of IE and outcome in nine series

Author, yr (reference) Setting, country	No. of IE	Patients with CNS complications, n (%)	Embolic events, n, (%)	Overall mortality (%)	Mortality of patients with CNS complications (%)	Cardiac surgery in patients with CNS complications, n (%)
Salgado, 1989 [47]	175	64 (36.5)	27 (42)	13.6	20.6	NR
One institution USA						

Roder, 1997 [40]	260	91 (35)		56	74	81 (89)
63 hospitals, Denmark						

Heiro, 2000 [3]	218	55 (25)	23 (42)	14	24	15 (27)
One institution Finland						

Anderson, 2003 [16]	707	68 (9.6)	49 (72)	NR	52 (1-yr)	13 (19)
One referral center USA						

Mourvillier, 2004 [7]	228	84 (37)	31 (37)	45 (in-hospital)	57	104 (46)
2 referral centers						
France						

Ruttmann, 2006 [30]	214	65 (30)	61 (94)	21	17 (median follow-up: 5.9 yr)	65 (100)
Cardiac surgery						
Austria						

Corral, 2007 [42]	550	71 (13)	42 (60)	11	34	26 (41)
One institution						
Spain						

Thuny, 2007 [8]	496	109 (22)	80 (73)	16 (6-mo)	22 (6-mo)	63 (58)
2 referral centers				19 (1-yr)	25 (1-yr)	
France				31 (5-yr)	38 (5-yr)	

Sonneville, 2011 [13]	198	108 (55)	79 (73)	57 (3-mo)	58 (3-mo)	53 (49)
23 ICUs						
France						

NR, not reported.

## Mechanisms, risk factors, and clinical patterns

Neurologic complications of IE can arise through the following mechanisms, frequently associated in the same patient: occlusion of cerebral arteries by emboli derived from endocardial vegetation; cerebral hemorrhage; infection of the meninges; brain abscess; and mycotic aneurysms. Sepsis-related encephalopathy, defined by acute confusional state or delirium, with fluctuation of vigilance also may contribute to neurologic manifestations of IE, especially in patients with *S. aureus *infection. Neurologic complications are a hallmark of left-sided abnormalities of native or prosthetic valves. CT scan is the most easily feasible neuroimaging in critically unstable patients. However, MRI is more sensitive and when performed should follow a standardized protocol that includes b1000 diffusion, T2* gradient recalled imaging, T2 fluid-attenuated inversion recovery (FLAIR)-weighted sequences, and three-dimensional T1 postcontrast [12].

## Cerebral emboli

Cerebral emboli result from dislodgment or fragmentation of cardiac vegetations, followed by vessel occlusion; this results in various degrees of ischemia and infarction, depending on the vessels and the collateral blood flow. Occlusion of cerebral arteries, with either stroke or transient ischemic attack, accounts for 40% to 50% of the CNS complications of IE [2]. More than 40% of cerebral emboli affect the middle artery. Among 198 ICU patients with left-sided IE, 108 experienced a total of 197 neurologic complications and ischemic stroke accounted for 40% of these episodes [13]. The main risk of neurologic complications is the absence of appropriate antibiotic therapy. Most neurologic complications are already evident at the time of hospitalization or develop within a few days. The probability of developing these complications decreases rapidly once antimicrobial therapy has been started. In the ICE-PCS study, the crude incidence of stroke in patients receiving appropriate antimicrobial therapy was 4.82/1,000 patient days in the first week of therapy and decreased to 1.71/1,000 patient days in the second week. This rate continued to decline with additional therapy [14]. Moreover, recurrent neurologic events, although possible even late, are uncommon. The localization of the infection has been found to influence the occurrence of neurologic events in some but not all studies, with a higher risk in patients with mitral valve vegetation [15]. Obviously, patients with large vegetations, measuring >10 or >15 mm and those with mobile vegetations are at increasing risk for embolism [15-17]. When neurologic complication rates were assessed as a function of the causative agent, the frequency of CNS involvement was two to three times higher with *S. aureus *than with other pathogens [3]. However, in IE caused by less frequent pathogens, such as *Streptococcus agalactiae *and fungi, the incidence of emboli is high and is explained by the large size of the vegetations [18,19].

**Figure 1:**
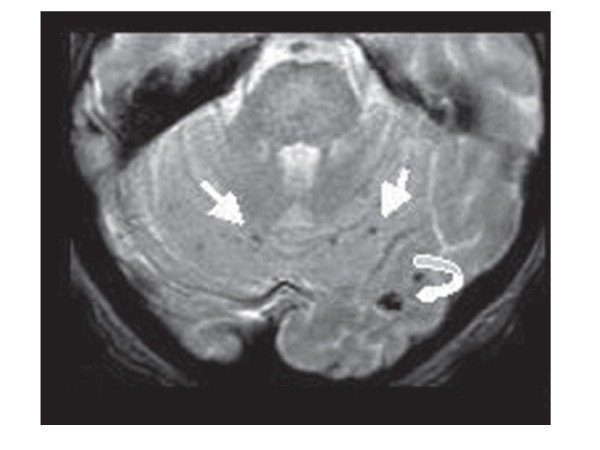
T2*-weighted gradient echo image. Multiple cerebellar microbleeds in a patient with infective endocarditis. (Reprinted from reference 12 with permission).

Emboli may cause a wide variety of clinical symptoms and signs, including impaired consciousness or focal deficits, depending on their size, location, and number. When systematic MRI is performed, large systematized and small ischemic lesions are seen in one third and two thirds of embolic episodes, respectively (Figure 1) [12].

## Cerebral hemorrhage

**Figure 2:**
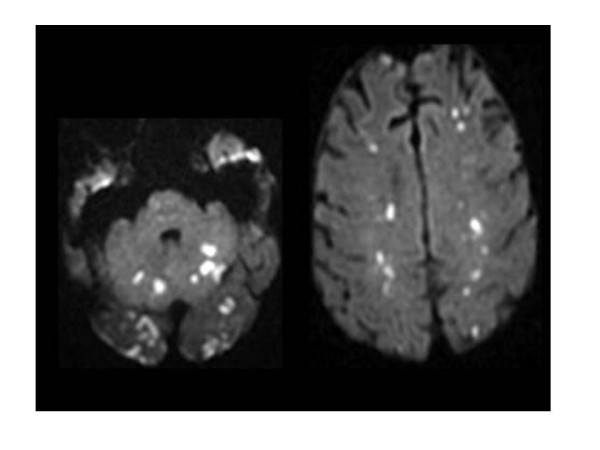
**Diffusion-weighted magnetic resonance imaging**. Acute hyperintense ischemic strokes in both hemispheres and in vertebro-basilar territories in the same patient (Reprinted from reference 12 with permission).

Cerebral hemorrhage accounts for 12% to 30% of neurologic complications of IE and even 29% of all neurologic complications in critically ill patients with IE [13]. They may be the result of different mechanisms. Transformation of ischemic infarcts caused by septic emboli is involved in approximately one third of patients with cerebral bleeding, either at the early phase of emboli or later. A recent case-control study, using diffusion-weighted MRI has revealed a high incidence of very small foci of hemorrhage. Cerebral microbleeds were observed in 57% of patients with IE compared with 15% of control subjects (Figure 2) [20]. These lesions may reflect a subacute microvascular process leading in some cases to the development of intracranial mycotic aneurysms (ICMA) on distal or pial arteries. Because of the strong association found between IE and cerebral microbleeds, the authors raised the question of additional diagnostic value of these lesions for IE.

Brain hemorrhage is more frequent during the bacteremic phase of *S. aureus *IE and is made more likely by severe thrombopenia and anticoagulant therapy [7]. Other mechanisms of bleeding are ruptured intracranial mycotic aneurysms and septic erosion of the arterial wall without a well-identified aneurysm. The latter complication is mainly seen in patients with *S. aureus *IE. Cerebral hemorrhage may be the first manifestation of IE and should be suspected in a febrile patient with sudden coma and/or neurologic deficit.

## Intracranial mycotic aneurysms

ICMA are relatively rare, accounting for less than 10% of neurologic complications of IE. They usually result from septic embolization to the vasa vasorum or to the intraluminal space of the vessel itself. Septic emboli are responsible for an inflammatory lesion starting on the adventice surface and ultimately destroying the intima. ICMA are multiple in 25% of the cases and are mostly located in the distal branches of the middle artery. Streptococci and to a lesser extent *S. aureus *are responsible for most ICMA. Nonruptured ICMA are responsible for fever, headache, seizures, and focal deficit. Patients with ruptured ICMA have sudden arachnoid or intracerebral bleeding, associating decreased level of consciousness, intracranial hypertension, and focal deficit. Rupture generally occurs at the early phase of IE, but in some patients, especially those with streptococcal IE, rupture may observed during antibiotic course of even after the end of therapy. CT-scan angiography and MR angiography are of equal value to detect ICMA >5 mm. Although conventional angiography may still be useful for the detection of very small ICMA, two-dimensional and three-dimensional helical CT also have high sensitivity [21].

## Meningitis and brain abscess

Meningitis or sterile inflammatory reaction to infection or brain ischemia or hemorrhage occurs in 2% to 20% of patients with IE and up to 40% of those with neurologic complications. In most cases, except in the rare cases of *Streptococcus pneumoniae *IE, the cerebrospinal fluid (CSF) is not purulent and the presence of pathogens is very transient. A typical ICU candidate has an acute febrile and toxic illness with heart murmur, petechiae, and meningeal signs. CSF examination finds moderate pleocytosis and Gram-positive cocci. Blood cultures yield *S. aureus*, and echocardiography confirms left-sided IE.

Brain abscess is considered to be a rare complication of IE but was observed in 14 (13%) of 108 critically ill patients with IE [13]. Although less than 5% of patients with brain abscess have IE, this complication should be suspected in the absence of obvious source and when multiple abscesses are present. Most brain abscesses observed in the setting of IE are caused by *S. aureus*.

## Consequences of neurologic complications on management of infective endocarditis

Neurologic complications may have consequences on the management of patients with IE. Their presence can help diagnosis because, as peripheral manifestations of IE, they are minor criteria in the Duke classification. They also can affect medical therapy by changing the type and length of antibiotic or anticoagulant therapy. Moreover, neurologic complications may influence indications, timing, and type of cardiac surgery. Finally, they may require specific approach, such as interventional neuroradiology to treat ICMA.

## Specific management of neurologic complications of IE

### Cerebral emboli

General supportive care, including airway, ventilator support, supplemental oxygen, control of temperature, management of blood pressure, and control of glycemia, are nonspecific measures and have been detailed elsewhere [22]. The proper use of antithrombotic therapy has given rise to much controversy but there is now some consensus, which was summarized in 2009 by "The Task Force on the Prevention, Diagnosis, and Treatment of Infective Endocarditis of the European Society of Cardiology" [23]. There is no indication for the initiation of antithrombotic drugs (thrombolytic drugs, anticoagulant, or antiplatelet therapy) during the active phase of IE. In patients who are already receiving oral anticoagulant therapy, this treatment should be replaced by unfractionated heparin for at least 2 weeks with a close monitoring of activated plasma thromboplastin or the activated cephalin clotting time. Although initial experimental studies showed a beneficial impact of aspirin therapy on the risk of an embolic event in *S. aureus *IE, there is no convincing clinical data to support its use. Finally, a retrospective cohort study of 600 adult patients with a diagnosis of IE showed that embolic events and related morbidity occurred significantly less in those who received prior, continuous daily antiplatelet therapy [24]. Therefore, interruption of antiplatelet therapy is not recommended in the absence of bleeding [23].

### Cerebral hemorrhage and intracranial mycotic aneurysms

Along with supportive measures, interruption of all anticoagulation is recommended, but in patients with mechanical valve unfractionated heparin should be reinitiated as soon as possible [23]. Treatment of ICMA remains a controversial issue because of the relative rarity of these lesions. Unruptured ICMA should be followed by serial imaging, because most will disappear with antibiotic therapy. If the ICMA is very large or enlarging despite antibiotics, or if the ICMA is ruptured, treatment will differ according to its location and the presence or not of masse effect. Endovascular therapy [25] should be considered when there is no mass effect and if the ICMA is located in a noneloquent territory. In contrast, in the presence of mass effect or location in an eloquent neuronal territory, neurosurgery is probably the best choice [26].

### Meningitis and brain abscesses

The presence of meningitis should not modify recommended antibiotic regimens because the first goal is to obtain clearance of pathogens from blood cultures. In patients with *S. aureus *brain abscesses, the addition to the standard regimen of a molecule penetrating brain parenchyma, such as a fluoroquinolone or rifampin, may be warranted. In most cases, patients have small and multiple abscesses that do not require surgery.

## Consequences of neurologic complications on cardiac surgery

In recent series [6,27], 48% to 50% of patients (up to 75% in specialized medical-surgical centers) undergo valve replacement during the acute phase of IE (i.e., before the completion of antibiotic treatment). In many studies, but not all, surgery is independently associated with a lower risk of mortality. Patients who benefit the most from cardiac surgery are those operated on for heart failure caused by severe aortic or mitral regurgitation, fistula into a cardiac chamber or valve obstruction [5]. Other indications are uncontrolled infection and prevention of embolism in high-risk patients. However, in patients with neurological complications, the safety of cardiopulmonary bypass has been controversially debated for years. Anticoagulation during cardiac surgery may increase the risk of hemorrhagic transformation of an asymptomatic ischemic stroke. Moreover, episodes of hypotension during procedure might exacerbate a pre-existing ischemic brain lesion. Finally, the need for anticoagulation in patients with mechanical valves increases the risk of cerebral bleeding. However, after a neurological event, most patients still have at least one indication for cardiac surgery. From studies published during the mid 1990s [28,29], an interval of at least 2 weeks between an embolic event and cardiac surgery was recommended. Several recent studies have challenged this statement and their results suggest that early cardiac surgery, when indicated, is possible even after a neurologic event. In a consecutive series of 214 patients undergoing cardiac surgery for IE, 61 had computed tomography- or magnetic resonance imaging-verified stroke. In those patients, early surgery (median 4 days) was not associated with more new neurologic events (3.2%) compared with late surgery, and the percentage of complete recovery was similar. However, in the case of middle cerebral artery stroke, recovery was only 50% and was significantly lower compared with non-middle cerebral artery stroke. Moreover, in comparison with non-stroke patients, the age-adjusted perioperative mortality risk was 1.7-fold higher and long-term mortality risk was1.23-fold higher in stroke patients [30]. Two other studies showed that urgent cardiac surgery in patients with embolic events was feasible with worsening of neurologic status in 6% and 0%, respectively [8,10]. Moreover, in a series of 48 patients with prosthetic valve IE, survival was better in patients operated within 8 days of diagnosis compared with those operated later [31]. In addition, the risk of neurologic deterioration after cardiac surgery in patients with silent neurological complications is probably very low [8]. Neurologic recovery depends on preoperative status, with a percentage of good recovery of 80% when National Institute of Health Stroke Score (NIHSS) is <9 but only 35% in patients with NIHSS >15 [30]. Among 108 ICU patients with neurologic complications of IE, 52 underwent cardiac surgery (median 10 days) at the acute stage of IE. Ten (19%) experienced new neurologic events after cardiac surgery and 33 (63%) survived, most with good functional outcome [13]. Finally, among 1,552 patients with native valve IE included in the ICE-PCS, cardiac surgery was found to confer a survival benefit among several groups of patients, including those with stroke [32].

The following recommendations have been made by "The Task Force on the Prevention, Diagnosis, and Treatment of Infective Endocarditis of the European Society of Cardiology": 1) after a silent cerebral embolism or transient ischemic attack, surgery is recommended without delay if an indication remains; 2) after a stroke, surgery indicated for heart failure, uncontrolled infection, abscess or persisting high emboli risk, should not be delayed. This recommendation does not apply to comatose patients; and 3) after intracranial hemorrhage, surgery must be postponed for at least 1 month (see also Table 2) [23].

**Table 2:** Cardiac surgery in ICU patients with IE and neurologic complications

Surgery possible if required	Surgery to be delayed or contraindicated
Heart failure, uncontrolled infection, abscess, high embolic risk	

Silent neurologic complications (CT scan, MRI)	Severe comorbidities

Transient ischemic attack	Severe septic shock

Stroke	Stroke and coma or extensive neurologic deficit

Microbleeds or very small hemorrhagic lesions	Intracranial hemorrhage (other than microbleeds or very small hemorrhagic)

Meningitis	Meningitis and coma (rare)

Brain abscess	Brain abscess associated with intracranial hypertension

Small ICMA	Very large or enlarging ICMA

## Impact of neuroimaging in management of patients with neurologic complications

No study has evaluated the effect of systematic neuroimaging on clinical decisions in ICU patients with IE. Cerebral MRI with angiography performed up to 7 days after admission led to a modification of therapeutic plans, including surgical plan modifications, for 24 (18%) of 130 patients mostly non-ICU patients [12]. However, because silent neurologic complications do not alter outcome, the role of systematic neuroimaging should be further evaluated. Although the majority of neurologic events are already present at ICU admission [13], some may occur later. Because clinical modifications may be difficult to evaluate in those ICU patients who are sedated, systematic CT scan or MRI should be performed when cardiac surgery is considered.

## Consequences on outcome of neurologic complications of IE

The overall in-hospital mortality of IE was 18% in the large, contemporary, and multinational ICE-PCE study [6]. This figure includes all types of IE and needs to be refined according to different categories of disease. Another recent cohort of 513 patients with complicated, left-sided native valve IE had a 6-month mortality rate of 26% [5]. Two studies found mortality rates for prosthetic valve endocarditis of 33% and 22%, respectively [27,33]. In the international ICE-PCE collaborative study, healthcare associated native valve endocarditis were associated with higher in-hospital mortality (25%) compared with community-acquired endocarditis (13%) [34]. Survival of ICU patients with IE is even lower. Among 228 patients with IE referred to two ICUs in a referral center, the in-hospital mortality rate was 45% [7]. It was 57% at 3 months in a multicenter study that involved 198 critically ill patients with IE [13], and 54% in 33 other ICU patients [35].

Neurologic complications may alter directly the outcome, increasing mortality and morbidity or indirectly by contraindicating early cardiac surgery. Indeed, in ICU patients with IE, mortality reached 88% in patients who were denied surgery, partly because of neurologic complications, despite a validated indication. Early surgery (within 7 days of diagnosis) was recently shown to increase free-event survival [36,37]. Many, but not all, studies showed that neurologic complications are associated with increased mortality during IE [3,6,16,38-43]. However, results of recent studies have provided more precise information with regard to the impact of neurologic events on outcome. First, patients with only silent or transient stroke had a better prognosis than patients with symptomatic events [8]. The former have mild or moderate brain lesions, which allow early surgery to be performed with a low operative risk. Second, more than the neurologic event *per se*, a better predictor of mortality is neurologic dysfunction, which is associated with location and extension of brain damage. In non-ICU patients, two studies [5,8] showed that impaired consciousness, evaluated by the Glasgow Coma score [44] or clinically, was a predictor of neurologic mortality. In ICU patients, survival of patients with or without neurologic events was not different, whereas there were more deaths in patients with neurologic failure defined by a neurologic Sequential Organ Failure Assessment score >2 [13], which corresponds to a Glasgow Coma score <10 [45]. Third, patients with severe neurological impairment and those with brain hemorrhage have the worse outcome. Besides mortality, neurologic recovery is a main concern. Among 106 ICU patients with neurologic complications assessed at follow-up (3.9 [3-8.5] months), only 31 (29%) had a modified Rankin Scale score <3 (ability to walk without assistance) [13]. Like mortality, neurologic outcome depends on the severity of brain damage as suggested by a study conducted in 68 patients with neurologic complications of IE. Full neurologic recovery was observed in 78% of patients with NIHSS at admission of 4-9 but in only 33% when the score was >15 [29].

## Conclusions

Neurologic complications are frequent in IE patients who require ICU admission. They contribute to a severe prognosis, especially in the case of neurologic failure. Improvement of outcome requires a multidisciplinary approach to optimize medical treatment and decision-making concerning valve surgery. This multidisciplinary approach has been shown recently to improve outcome in patients with endocarditis [46].

## Competing interests

The authors declare that they have no competing interests.

## Authors' contributions

RS drafted the manuscript. The manuscript was revised for important intellectual content by BM, LB and MW. All authors read and approved the final manuscript.
